# Behavioral algorithms and neural mechanisms underlying odor-modulated locomotion in insects

**DOI:** 10.1242/jeb.200261

**Published:** 2023-01-13

**Authors:** Samuel P. Wechsler, Vikas Bhandawat

**Affiliations:** School of Biomedical Engineering, Sciences and Health Systems, Drexel University, Philadelphia, PA 19104, USA

**Keywords:** Circuit, Insect, Search, Neural mechanisms, Odor tracking, Olfaction

## Abstract

Odors released from mates and resources such as a host and food are often the first sensory signals that an animal can detect. Changes in locomotion in response to odors are an important mechanism by which animals access resources important to their survival. Odor-modulated changes in locomotion in insects constitute a whole suite of flexible behaviors that allow insects to close in on these resources from long distances and perform local searches to locate and subsequently assess them. Here, we review changes in odor-mediated locomotion across many insect species. We emphasize that changes in locomotion induced by odors are diverse. In particular, the olfactory stimulus is sporadic at long distances and becomes more continuous at short distances. This distance-dependent change in temporal profile produces a corresponding change in an insect's locomotory strategy. We also discuss the neural circuits underlying odor modulation of locomotion.

## Teaser

**Summary:** This Review explores the behavioral and neural mechanisms that insects use to locate food and mates using their sense of smell.

## Introduction

A question that we often get from laypeople and expert scientists alike is how sharks find their victim a mile away. The myths that sharks can detect blood and home in on their prey from large distances remain persistent despite efforts both in popular science (https://www.youtube.com/watch?v=ugRc5jx80yg) and in peer-reviewed work to dispel them (Gardiner et al., 2012; Meredith and Kajiura, 2010). Sharks do possess a nervous system that is exquisitely sensitive to chemicals in blood and can likely detect the blood of potential prey from a mile away. However, tracking resources based on their smell (odor tracking) is more challenging than just detecting an odor, because odor gradients are not preserved beyond the immediate vicinity of the odor source. The mechanisms of odor dispersal allow odors to be detected at long distances without providing directional cues because animals typically experience concentrated patches of odor followed by clean air (Box 1). This difference between detection and tracking is best quantified in the context of the champion smellers in the insect world – male moths. Males of many moth species can detect a single molecule of the female pheromone (Kaissling, 1986). However, this exquisite sensitivity does not allow them to track down females from a kilometer away as suggested by earlier studies (Bossert and Wilson, 1963; Collins and Potts, 1932). Later work has demonstrated that it is hard for moths to locate females even 80 m away (Elkinton et al., 1987).

**Box 1. The role of odor dispersal in odor tracking**

Odor dispersal, a topic covered in detail in other reviews (Capelli et al., 2013; Celani et al., 2014; Elkinton et al., 1984; Murlis et al., 1992; Riffell et al., 2008), is essential to understanding odor tracking. There are two mechanisms by which pheromones released by a small odor source (red dot in the figure) such as a female gypsy moth can disperse: diffusion, and advection and convection (top panel is a snapshot of odor distribution; gray patches represent odor concentration). Diffusion is a process in which the odor molecules move down a concentration gradient. Diffusion rates are so low that it can be discounted as a mechanism for odor dispersal beyond a few centimeters from the odor source (Riffell et al., 2008). Much of the dispersal occurs through advection and convection, processes by which a mass of air moves owing to spatial differences in air density, pressure and temperature, carrying odor molecules with it. This mode of dispersal has two consequences for odor tracking. First, odors move in packets such that local odor concentration is above the detection threshold for long distances from the odor source (bottom panel); this makes odors the first source of information about a resource. Second, the distribution of odor packets in space might be informative about the location of the odor source (Boie et al., 2018), but is not a strong predictor of source location in a dynamic environment. Therefore, odors provide information about objects from afar without providing a roadmap to the object that other senses such as vision might. Near the odor source, the odor pulses that an animal experiences are no longer transient; they become continuous (note the consistently gray region adjacent to the odor in the top panel). In one set of measurements, at 4 m from the source, the stimulus was present 75% of the time, while being present only 20% of the time at 40 m (Baker et al., 2018); similar observations have been made by others (Murlis et al., 2000). This change drives the change in behavior observed as the insects come close to the odor.
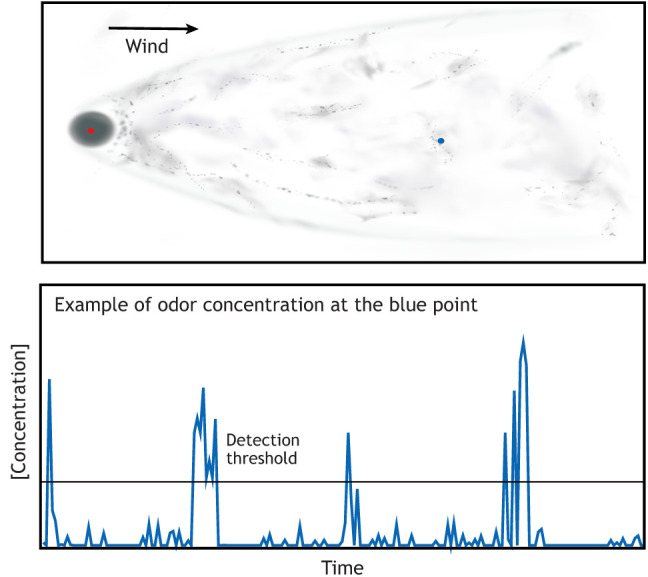

Nevertheless, odor tracking is ubiquitous in the animal kingdom, albeit not over kilometers, and underpins many behaviors essential to an animal's survival. How does an animal go about finding the source of an odor in the absence of directional cues from odor concentration? The best source of directional information is wind direction. When wind direction is constant, flying upwind upon odor contact is an excellent strategy because the odor source is likely to be upwind. However, in the real world, wind direction is rarely constant (David et al., 1982), which means that the present upwind direction and the direction of the odor source are not always the same (Brady et al., 1989).

Thus, the problem confronting any animal performing odor tracking is how the sporadic detection of odor can be efficiently used to get closer to the source of the odor.

Even under the best of circumstances, odor tracking itself only leads the animal to the vicinity of the source, and not directly to the source itself. There are various reasons for this. In the case of the moth, likely owing to the eddies under the tree, odor tracking cannot direct the insect to the source, just to the right tree (Charlton and Cardé, 1990); often not even that (Doane, 1968). Similarly, odor plumes emanating from a mammal can be as large as the entire animal, but a mosquito will still feed preferentially from specific body parts (De Jong and Knols, 1996). Long-range odor tracking is replaced by a different strategy – local search – near the source of the odor. For example, once odor tracking leads a male moth to the right tree, the moth flies vertically in the immediate vicinity of the tree, lands on the tree trunk and walks the last few centimeters to the female (Charlton and Cardé, 1990). Over short distances near the female, visual cues might play a role (Charlton and Cardé, 1990; De Jong and Knols, 1996; Doane, 1968). In some cases, such as flower feeding by moths, a conjunction between olfaction and vision is necessary for successful feeding (Raguso and Willis, 2002).

So far, we have discussed the challenges of finding the location of an odor. Another equally difficult problem that animals must contend with is identifying an odor. The olfactory environment is complex and rich (Herrmann, 2011). Odors from the resource that an insect is seeking are mixed in with odors – sometimes closely related ones – from other sources. Insects must discriminate the odors from the resource from this complex mix (Riffell et al., 2014). The behavior towards a given odor is also highly dependent on the state of the animal, such as feeding or mating status.

**Fig. 1.**
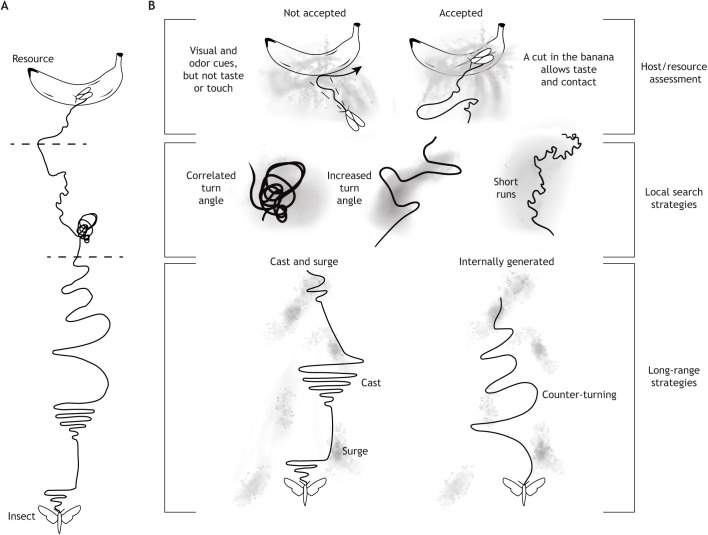
**Insects employ distance-dependent locomotor strategies.** (A) An insect can sense odors a long distance away. Insects employ distance-dependent strategies to find the resource, assess it and accept it. Here, an insect flying to the odor source comes near it and changes its strategy to local search before assessing the resource. Some of the behavioral strategies are shown. (B) The behavioral strategy changes, in part, because the odor profile changes from patchy (gray shading) at long distances to continuous near the odor source. In response, the behavioral strategies are different as well. Far from the odor source, insects use long-range strategies. Two of these strategies are caste-and-surge and internally generated counter-turning. When using the caste-and-surge strategy, insects surge upwind on encountering an odor, and perform frequent turns perpendicular to wind direction after losing the odor. Internally generated counter-turning is similar to caste-and-surge; the main difference is that behavior is driven by an internal program and not by odor encounters. Odor detection activates this behavior. Closer to the odor source, the insect aims to stay close to the odor source through a variety of local search strategies. These changes in behavior – including correlated turning, increased turn angle, and short runs punctuated by changes in direction – have the effect of keeping the insect close to the odor source. Finally, insects assess the resource and choose to accept or reject it. This assessment depends on other modalities including vision, taste and touch.

In summary, odor modulation of locomotion is not a single behavior optimized to find the source of odor. Rather, it is a suite of behaviors that together ensure that animals can find and exploit resources critical to their survival (Fig. 1). Odor-guided locomotion requires exquisite sensitivity to multiple sensory systems, neural circuits to process and integrate sensory information, spatial memory, behavioral flexibility and the ability to act with incomplete information. Insects possess all these capabilities.

In this Review, we will consider behavioral algorithms (see Glossary) underlying odor modulation of locomotion in insects and the neural circuits underpinning this behavior. We draw on research performed in various insects, but note that most of the work has done in moths, cockroaches and flies. This Review is divided into three sections. We start by reviewing behavioral algorithms that underpin different aspects of an insect's odor-tracking behavior, followed by a review of how the behavioral algorithm is implemented in the insect's brain. Finally, we review the neural circuits underlying odor identification and discuss future research avenues.

**Glossary**

**Behavioral algorithm**

A set of rules for selecting an appropriate action or sequence of actions from a set of pre-established behaviors to accomplish a given task.

**Generative model**

A model that can generate new data. Here, it means a behavioral model that generates new locomotion trajectories that can be compared with actual data to assess whether model trajectories are consistent with empirical data.

**Glomerulus**

A clustering of nerve endings. Here, it refers to the region within the antennal lobe where olfactory receptor neurons that express the same olfactory receptor project into.

**Laminar plume**

Airflow moves smoothly in a regular path, producing a continuous ribbon of odor filament projecting from the source location.

**Multimodal integration**

Integration of information from different sensory modalities.

**Neuropil**

An area within the nervous system where there is a high density of synapses but relatively few cell bodies.

**Odor-gated anemotaxis**

Turning upwind when a salient odor is encountered.

**Olfactory receptor neurons (ORNs)**

Receptors housed in specialized hairs called sensilla within the antennae that are activated in response to airborne odorants.

**Patch border**

The point between an odor plume and odorless space where the concentration of odor is sufficient to pass a detection threshold.

**Protocerebral**

Pertaining to the protocerebrum, a prominent neural structure within the insect brain that contains important neuropils such as the mushroom body and central complex.

**Resource patch**

Resources are not distributed randomly. They are distributed in clusters called patches. Sensory stimuli including odor, tastants or visual stimuli can signal a resource patch.

**Sensorimotor reflexes**

The modulation or initiation of behaviors in response to a specific sensory cue.

**Turbulent plume**

Fluctuating, irregular airflow causes odor filaments to be dispersed amongst intermittent pockets of odorless space.

**Turn bias**

The propensity to turn in the same direction, say, clockwise.

**Visually guided anemotaxis**

Maintaining a fixed trajectory with respect to the wind direction using visual cues for steering. This behavior is important during flight.

## Behavioral algorithms underlying odor modulation of locomotion

Understanding behavioral algorithms underlying odor modulation of locomotion is a formidable challenge; researchers have met this challenge with a range of behavioral paradigms (Box 2). We describe behavioral algorithms at two different spatial scales. We start with describing medium-range navigation to a source of odor. In this regime, the animal has detected an odor but does not know the source location and seeks to find this source. Then, we review near-range navigation during which the insect has either narrowed down the source considerably or has already found it and is taking the last few steps to engage with the source.

**Box 2. Studying odor-guided locomotion in the lab**

Odor-guided locomotion is a challenging problem as the complexity and diversity of the odor landscape experienced by insects in nature is difficult to replicate in the lab. Furthermore, even in simplified laboratory experiments, it is difficult to quantify when the animal encountered an odor, making it difficult to evaluate the animal's underlying strategy. Inferring strategy from an animal's circuitous walking or flight paths is itself a daunting problem. Despite these challenges, much progress has been made in understanding the behavioral algorithms at play during odor-modulated locomotion by performing experiments in simpler behavioral arenas that, with some exceptions, fall into three types. In the first type, insects navigate towards an odor source in a laminar plume. These experiments are performed in a wind tunnel at low wind speeds such that there is a small cylinder of odorized region within the tunnel. The second type of experiment uses similar methods, but with turbulent rather than laminar plumes. These turbulent plumes still do not capture the complexity of real-world plumes because the wind direction is also constant, and much of the spatial scales of turbulence observed in nature are too large to be observed within a wind tunnel. Finally, the third type of experiments is conducted in still air without any wind.

### Medium-range navigation towards an odor source

The presence of a resource is often first signaled by the detection of odor, i.e. the resource is smelt before it is seen or touched – and the animal's initial response is influenced by odor alone. As the animal approaches the source, its behavior is affected by multi-modal integration. The distance at which behavior becomes multimodal depends on the species and the environment. Moreover, the processes described here are not specific to a single mode of locomotion, as the effect of odors on behavior is similar during both flight and walking (see ‘Local search’ section for details).

#### The reflexive cast-and-surge program

In both walking and flying insects, there are two conserved motor programs that aid in medium-range navigation to an odor source: the reflexive cast-and-surge program and the internally driven counter-turning. The reflexive cast-and-surge program consists of upwind locomotion or odor-gated anemotaxis (see Glossary); many insects either show little directional preference or walk/fly downwind in the absence of odor, but will travel upwind in the presence of odor (Alvarez-Salvado et al., 2018; Budick and Dickinson, 2006; Willis and Arbas, 1998; Willis and Avondet, 2005; Wolf and Wehner, 2000). Odor-gated anemotaxis (Kennedy and Marsh, 1974) consists of a two-component motor program where both components are sensorimotor reflexes (see Glossary) (Fig. 1). The first component is surge, in which contact with an odor results in rapid upwind movement; surge can be phasic or tonic (Budick and Dickinson, 2006) depending on the species (Fig. 1A). The second component, cast, which occurs upon loss of odor, results in a cessation of upwind progress and execution of turns. In many, but not all, insects, these turns gradually widen, and between each turn the insect travels perpendicular to the wind direction.

The cast-and-surge strategy and its origin as a sensorimotor reflex was first proposed by Baker and colleagues (Baker, 1990) based on a clever deduction; they realized that responses to pheromone (Baker and Haynes, 1987) and odor encounter rate (Baker and Haynes, 1989) had similar frequency. Measurement of odor contact during free flight in two moth species (Mafra-Neto and Cardé, 1994; Vickers and Baker, 1994) showed that contact with female pheromone led to an upwind surge with a ∼200 ms delay that lasted approximately 500 ms and terminated in a cast. Since these pioneering studies, the cast-and-surge strategy has been demonstrated in other flying insects (Dekker and Cardé, 2011; Thiery and Visser, 1986; van Breugel and Dickinson, 2014), and also during walking in both cockroaches (Bell and Tobin, 1981) and in *Drosophila* (Alvarez-Salvado et al., 2018)*.* An iterative cast-and-surge strategy will bring the insect closer to the source of odor and also explains the difference in behavior under different stimulus conditions. In laminar plumes (see Glossary), the moth turns frequently and flies crosswind because each surge takes the insect out of the odor, and contact is only made after the moth turns around (Mafra-Neto and Cardé, 1994). In turbulent plumes (see Glossary), where the contact with odors is intermittent, the moth's trajectory is straighter owing to the fact that each contact with the odor results in a surge that is barely extinguished before the next odor contact is made, resulting in another upwind surge (Mafra-Neto and Cardé, 1994; Mafra–Neto and Cardé, 1995). Strikingly, when pheromones are pulsed at a high enough frequency, even the tracks in a ribbon plume become straight because each surge ends in another odor stimulation, leading to another surge and completely extinguishing turns (Mafra-Neto and Cardé, 1994, 1995, 1996).

#### The internally driven counter-turning

The internally driven counter-turning requires odor for its expression (‘gating’) but is not a direct response to odor encounters; odors play a permissive rather than an instructive role. This motor program also has two components that are roughly analogous to cast and surge but have different mechanisms (Baker et al., 1984; Kennedy and Marsh, 1974; Willis and Arbas, 1991; Wright, 1958) (Fig. 1). Equivalent to surge but not resulting from a direct contact with odor, the insect has straight flight segments during which it maintains constant ground speed and orientation in relation to wind direction (David and Kennedy, 1987; Haynes and Baker, 1989; Marsh et al., 1978; Von Keyserlingk, 1984; Willis and Baker, 1994; Willis et al., 1991), reflecting visually guided anemotaxis (see Glossary). These straight segments are interrupted by crosswind turns that occur at remarkably regular intervals (David and Kennedy, 1987; Haynes and Baker, 1989; Von Keyserlingk, 1984), suggesting that they are generated internally (Willis and Arbas, 1991) rather than being a consequence of discrete odor encounters. Odors also modulate this program: an increase in the number of odor encounters results in decreased speed (Baker and Haynes, 1987; Kennedy, 1983; Marsh et al., 1978; Willis and Baker, 1994). In some moths, the frequency of counter-turning also increases as the moth approaches the odor source (Kennedy, 1983; Kuenen and Baker, 1982; Willis and Arbas, 1991). Because speed decreases and the frequency of counter-turning increases as the insect approaches the source of odor, the crosswind excursions become smaller, giving the impression that the insect is homing in on the odor source (Marsh et al., 1978). In contrast, decreasing odor encounters leads to wider casts (David and Kennedy, 1987).

#### The contribution of motor programs to finding an odor source

The cast-and-surge motor program and the internally generated counter-turning are similar and might appear to be just a single motor program. Some authors have made a distinction between them based on the characteristics of the cross-wind movement, which they classified as either zigzagging or casting, casting being movement perpendicular to wind direction without any upwind progress and zigzagging being movement with upwind progress (Kennedy et al., 1981; Preiss and Kramer, 1986). These differences could be real and significant; however, it is difficult to convincingly distinguish between the different mechanisms without quantifying the relationship between sensory stimulus and each turn – an important avenue for future research. Previous studies have emphasized the reflexive aspects of the tracking behavior over the internally generated program (Baker and Haynes, 1987; Baker and Vickers, 1997; Budick and Dickinson, 2006; van Breugel and Dickinson, 2014) because they have focused on turns immediately after an odor encounter. However, the most parsimonious interpretation of these studies is that the reflexive cast-and-surge strategy is superposed on top of the internally generated counter-turning, and both are necessary to explain an insect's overall behavior; experiments aimed at testing whether this interpretation is correct constitute a particularly fruitful line for future research.

Having both reflexive and internally driven counterturning would make odor tracking more robust. Tracking an odor plume, particularly in flight, is difficult. A recent study found that flies can only stay within a predictable, cylindrical plume for 500 ms (van Breugel and Dickinson, 2014). Similarly, sensory delays of 200 ms typically associated with cast-and-surge strategies imply that an animal is always reacting to the past and not the present. Errors and delays are not debilitating when the wind direction is constant, because turning would lead the insect back into the plume, as the insects exit the plume mostly because of misalignment with the upwind direction. However, in realistic plumes with variable wind direction and speed, turning back does not ensure odor encounter, and the likely existence of long intervals during which there is no odor contact makes an internally generated strategy necessary. A long-lasting strategy with frequent changes of direction is more likely to result in contact with odor because the insect will end up re-encountering the plume by chance. Slowing down as encounters become more frequent would increase the chance that insects would stay close to the plume; this slow down close to an odor source has been observed in flies (Saxena et al., 2018).

The idea that a reflexive strategy works well in predictable conditions and internally generated counter-turning performs better in a more unpredictable environment is supported by modeling studies (e.g. Belanger and Willis, 1996).

#### Other mechanisms in medium-range odor tracking

Another important conclusion from the Belanger and Willis (1996) study is that the known mechanisms of odor tracking did not come close to the performance of the actual moth, demonstrating that there are additional mechanisms at play. Precise control over odor stimulation, detailed analysis of an insect's tracks and generative models (see Glossary) to assess how well behavior is understood in walking *Drosophila* have led to the discovery of these mechanisms. A recent study, which took advantage of optogenetic stimulation to create a precise pattern of olfactory stimulation, showed that activating a fly's olfactory system did not change the fly's propensity to turn while exiting an odorized area (Tao et al., 2020). Rather, flies slowed down as they exited the odor plume, giving the impression that there is increased turning at the border; the turns made at the border of the odorized area were much larger. That study also found that there are kinematic changes associated with olfactory stimulation that cause the flies to slow down in the stimulus and increase its speed outside the stimulus region. Another recent study that replicated turbulent plumes with more precise stimulus control than in previous experiments demonstrated that the fly's behavior is much better modeled as stochastic than as a pure sensorimotor reflex (Demir et al., 2020). Moreover, that study showed that odor encounters modulated the stop-to-walk transition, an important movement characteristic. In flies, a recent study also found that odors affect multiple aspects of locomotion (Jung et al., 2015). Recent advances in machine vision and statistical techniques will help us to make progress in discovering mechanisms by which odors affect locomotion, and how the entire ensemble of mechanisms helps insects to approach the odor source.

### Local search near the odor source and harvesting the resource

The mechanisms described above operate when the insect is far from the odor source. Often, the insect's behavior changes close to the source: a male moth reacting to female scent, after flying upwind and reaching the right tree, performs vertical flights to find the correct landing spot, lands on the tree and performs a local search by walking, and finally makes contact with the female (Charlton and Cardé, 1990). A similar behavioral transformation is observed – this time in flight – as the moth approaches a flowering plant (Raguso and Willis, 2002). This time, the moth hovers over the flower. Mosquitoes, too, change their behavior as they approach their host. Far from the odor source (>10 m), it is driven primarily by detection of CO_2_, and close to the odor source (<10 m), it is driven by a combination of vision and odor (Van Breugel et al., 2015) before landing and searching. Sandflies land non-preferentially on their host – a mammal – but then move to a region with less hair, such as the ears or eyelid, to feed (Coleman and Edman, 1988). Even for insects that just walk, the strategy changes as the animal approaches the odor source (Wolf and Wehner, 2000). Regardless of whether the locomotion mode changes, there can be a behavioral switch. Both the nature of the behavioral change and where it occurs (how far from the odor source) depends on the species, environmental conditions, the density of available resources and other factors (Charlton and Cardé, 1990; Wolf and Wehner, 2005). In this section, we describe the behavior near the odor source; the insect's objective has changed from approaching the odor source to locating, assessing and utilizing the resource it signals.

One change is that the insect's locomotion strategy is altered into a local search strategy, likely in response to the stimulus becoming more continuous and/or other sensory modalities, such as vision and taste, that are also present, representing a resource patch (see Glossary) (Fig. 1). Local search in insects was first discovered in blowflies, which change their locomotion to a local search after feeding on sugar, and this was thought to be initiated by resource utilization (Dethier, 1957; Murdie and Hassell, 1973; Vinson, 1977). A similar local search pattern is also observed on encountering resource-specific cues such as food odors or sex pheromones (Jung et al., 2015; Sabelis et al., 1984).

Just like medium-distance navigation to the odor source, local search is not a single motor program but a constellation of mechanisms that result in the animal being restricted to a given area. One mechanism is looping (or spiraling), which involves an increase in the animal's turn rate, with the animal maintaining a turn bias (see Glossary) in a single direction, resulting in looping trajectories that bring the animal back to the same location, essentially circling the resource (Beevers et al., 1981; Sabelis et al., 1984). Another mechanism is a decrease in run length or in the distance between each subsequent stop. This has been observed in bumblebees (Heinrich, 1979) and honeybees in a patch of flowers (Schmid-Hempel and Schmid-Hempel, 1986), and in flies in response to odor alone (Jung et al., 2015) (Fig. 1).

A mechanism that has received particular attention is turning back into the resource patch when the patch border (see Glossary) is encountered. Unlike spiraling or changes in run length, turning back requires a sense of direction. Decreasing odor concentration can serve as a directional cue that can be sensed by simultaneously comparing concentration at two locations. Because olfactory receptors are present within the insect's antennae – elongated, jointed sense organs that are attached to the insect's head – comparison of odor concentrations across two locations to turn towards the side that experiences the higher concentration is possible (Borst and Heisenberg, 1982; Duistermars et al., 2009; Martin, 1965). Odor concentration at two locations can also be measured sequentially by simply walking to different locations (Bell and Tobin, 1982; Lockey and Willis, 2015); this computation requires short-term memory. Moreover, insects successfully turn at the border using a large increase in turn amplitude even when the patch abruptly ends and there is little scope for them to evaluate concentration (Sabelis et al., 1984; Waage, 1978). In *Drosophila*, a large decrease in speed is coupled with an increase in turn amplitude (Tao et al., 2020).

The local search mechanisms can be elicited by other sensory modalities such as gustation (Mayor et al., 1987; Nelson, 1977) or vision (Bell et al., 1983; Lawrence, 1982) alone, which suggests that local search mechanisms can utilize the sensory modality that provides the most salient stimulus. In contrast to changes in locomotor strategy, acceptance or rejection of a resource such as food, oviposition site or mate often requires a conjunction of multiple sensory modalities (Fig. 1). The synergism between vision and olfaction is important for locating the odor source and landing (Frye et al., 2003; Saxena et al., 2018; Stewart et al., 2010; Van Breugel et al., 2015; Vinauger et al., 2019). Similar multimodal interactions are observed in oviposition (Harris and Miller, 1982; Spencer et al., 1999), initiation of feeding (Raguso and Willis, 2002; Wheelwright et al., 2021) and courtship (Krstic et al., 2009; Pan et al., 2012).

As summarized in Fig. 1, odor modulation of locomotion involves distance-dependent locomotor strategies. At each distance, a whole suite of changes in locomotion characterizes changes in behavior. As described above, different insects employ these strategies to different extents, and the details of a given strategy would also differ from insect to insect. Unraveling all the behavioral strategies employed, how these strategies are deployed based on current sensory conditions and how differences in behavior between insects reflect adaptation to their ecological niche are all important avenues for future research.

## Neural mechanisms underlying odor modulation of locomotion

The behaviors described above require many computational abilities: one is to process and integrate information from different sensory modalities, called multimodal integration (see Glossary). Odor information is combined with wind direction and full-field visual signals, such as optic flow, to navigate towards the odor from large distances (Cardé and Willis, 2008). Near the odor source, visual recognition of small objects is combined with other sensory cues to land on the object if the animal navigates to the odor in flight (Raguso and Willis, 2002). Gustatory, visual and mechanosensory information is combined with olfactory information to decide whether to accept or reject the resource. A second ability is memory – both spatial and episodic. Spatial memory is required to keep track of one's position in space to direct the next movement, whereas episodic memory is necessary to recall past odor encounters and make decisions based on odor history (Ache et al., 2016; Baker et al., 2018; Pang et al., 2018). Finally, behavior depends on other circumstances, such as an animal's risk assessment and its own state and motivation. In the following sections, we will discuss these three abilities in insects, and how they aid or limit an insect's ability to locate and utilize resources. It is important to note that these neural circuits are conserved enough across insects (Ito et al., 2014; Martin et al., 2011) that, despite some differences, the basic computation and logic are similar; therefore, in discussing the role of different circuits, we draw on research across insects.

### Unimodal sensory processing of odors, wind and photons

The basic circuit that senses and processes olfactory information is described in Box 3. Odors are detected by olfactory receptor neurons (ORNs; see Glossary); a large number of ORNs converge onto a single second-order neuron called a projection neuron (PN).

**Box 3. Circuits for olfactory processing in insects**

Odor detection occurs in the olfactory receptor neurons (ORNs) present in the antennae and palps. Each ORN expresses one or a few odorant receptors (ORs); the number of receptors range from just 10 in some lice (Hansson and Stensmyr, 2011; Kirkness et al., 2010) to a few hundred in bees (Robertson et al., 2003). The ORs expressed in each ORN determine its odor response profile. In many (Schachtner et al., 2005) but not all insects, ORNs expressing a given receptor (different ORN classes in the figure) project to a single glomerulus, where they interact with second-order neurons called projection neurons (PNs). Approximately half of the PNs in *Drosophila* are themselves uniglomerular (uPN), and the other half are multiglomerular (mPN). A large majority of uPNs use acetylcholine, the major excitatory neurotransmitter in invertebrates; a minority use GABA as their neurotransmitter. The division of mPNs into excitatory versus inhibitory is more equal. The outputs of the antennal lobe are four different channels of information: excitatory (in magenta in the figure) and inhibitory uPNs (in green in the figure) as well as excitatory and inhibitory mPNs (Bates et al., 2020). The presence of these parallel pathways from the antennal lobe to higher brain centers is conserved across insect orders, but there are also important differences (Galizia and Rössler, 2010).
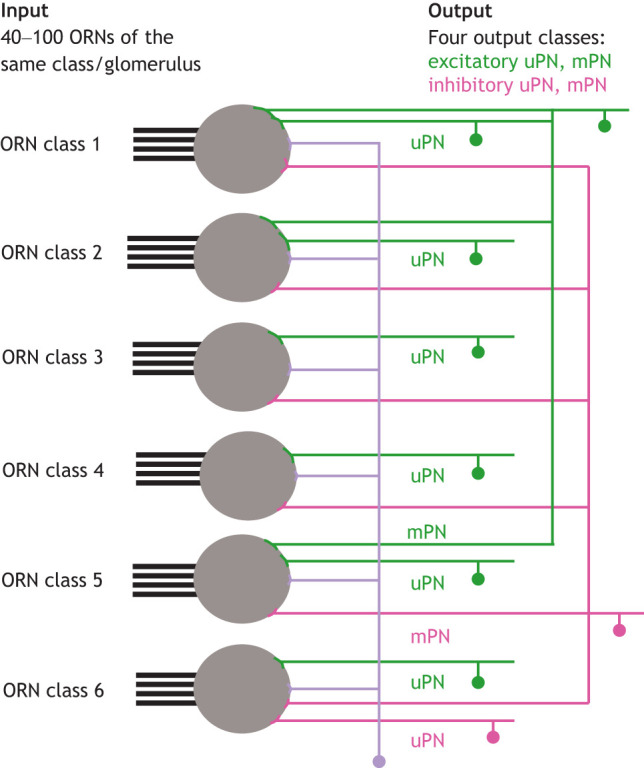

#### Convergence increases the sensitivity to odors

The sensitivity of individual ORNs and the convergence from ORNs to PNs allows insects to detect odors at low concentration with short latency. Estimates suggest that a single moth pheromone molecule can produce a change in firing rate in an ORN that is specific to pheromones (Kaissling, 1986). Even when ORNs are not specific to a single odor, they can still be sensitive to odors (Hallem and Carlson, 2006; Olsen et al., 2010). An insect's ability to detect odors is further enhanced through convergence from the ORNs to the PNs, which provides a mechanism for amplification (Kazama and Wilson, 2009). In *Drosophila*, 40 to 100 ORNs project to the same glomerulus (see Glossary); each ORN synapses on each uniglomerular PN (uPN) (Kazama and Wilson, 2009), which results in an amplification of weak odor responses (Bhandawat et al., 2007; Olsen et al., 2010). Convergence also shortens the latency to detect an odor, an important consideration when tracking odors in an ever-changing environment (Jeanne and Wilson, 2015).

There is additional circumstantial evidence that convergence is an important mechanism for increasing odor sensitivity (Hansson and Stensmyr, 2011). In many insects, the antennae are highly branched to accommodate thousands of pheromone-sensitive sensilla (Keil, 1989; Nishino et al., 2018), presumably to increase sensitivity. Moths also have a sexually dimorphic macroglomerular complex (Koontz and Schneider, 1987), a set of glomeruli that process sex pheromones, that is enlarged in males (Boeckh and Boeckh, 1979; Hansson et al., 1992); similar expansion is also observed in drosophilid flies (Kondoh et al., 2003). The increased glomerular size is likely related to an increase in ORN numbers, a phenomenon also observed for ORNs involved in the detection of other non-pheromonal volatiles. Two examples include the expansion of ORNs that detect a specific food source in the specialist *D. sechellia* compared with the generalist *D. melanogaster* (Dekker et al., 2006), and in mosquitoes (Syed and Leal, 2009).

#### Comparison of odor concentrations at different body parts

As discussed above, it is unlikely that an instantaneous concentration comparison between ORNs in different parts of the body such as the two antennae plays a large role in odor tracking over long distances. However, instant comparison appears to play a crucial role in trail tracking across the animal kingdom (Hangartner, 1967; Rajan et al., 2006; Takasaki et al., 2012) and is involved in determining the borders of a resource patch (Bell, 1985). Concentration comparison can be crucial under conditions in which there are sharp odor gradients, but it does not appear to be the only mechanism (Tao et al., 2020). There are several neural mechanisms that can extract and accentuate local concentration differences at the two antennae. In *Drosophila*, where most ORNs project bilaterally, the PNs can differentiate between ipsilateral and contralateral ORNs, likely based on the different axon lengths of the ipsilateral and contralateral ORN axons, which result in a time difference between signals from the two antennae reaching PNs (Gaudry et al., 2013). In both moths and cockroaches, a more elaborate architecture, whereby pheromone-related ORNs in different parts of the antennae project to small sub-regions of the glomerulus, exists to take advantage of different spatial patterns of odors (Nishino et al., 2018). PN responses, too, were responsive to the location of the odor stimulus on the antennae. This topographical arrangement appears to be maintained in higher-order olfactory circuits and, in principle, can create a map of instantaneous pheromone concentrations. Whether an instantaneous map of the local distribution of pheromone concentration (or other odors) is created and how these instantaneous comparisons are employed in driving behavior is an important avenue for future investigation.

#### Contribution of mechanosensation and vision to odor-guided behaviour

**Fig. 2.**
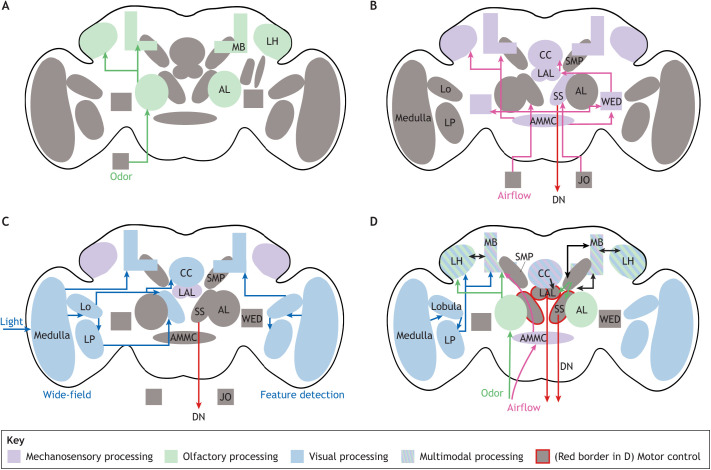
**Circuits underlying odor-guided locomotion.** (A) Regions of the brain important for olfactory processing (green). Odors are detected by neurons in the antenna. These neurons project to the antennal lobe (AL). Projection neurons from the antennal lobe project to mushroom body (MB) and lateral horn (LH). (B) Airflow (in magenta) information is also important for odor-guided behavior. Airflow is detected by the Johnston organ (JO) neurons in the antenna; through various intermediate centers, such as antennal mechanosensory and motor centers (AMMC) and Wedge (abbreviated as WED), these neurons connect to central complex (CC) neuropils to allow insects to orient themselves with respect to airflow. (C) Visual (in blue) information is also important for odor-guided behavior. Two parallel streams of visual information – wide-field information, such as that arising from motion, and feature detectors – are important for odor-guided behavior. The lobula (Lo) and lobula plate (Lp) are important visual processing centers. (D) Flow of information underlying odor-guided locomotion. Neuropils in green, blue and magenta are largely unimodal sensory processing centers that process olfactory, visual and mechanosensory information. Many central brain regions (marked with striped color) such as MB, LH and the superior medial protocerebrum (SMP) play an important role in multi-modal integration through connections from multiple sensory systems and recurrent connections between each other. Motor commands originate from the lateral accessory lobe (LAL) and from the superior slope (SS). Motor regions are marked with a red border. Descending neurons (DNs) carry motor-related information from the brain to thoracic ganglia (Lyu et al., 2022).

We will only discuss mechanosensation and vision briefly as these modalities have been covered in greater detail in other reviews (Borst et al., 2020, 2010; Krishnan and Sane, 2015; Silies et al., 2014). We will first discuss mechanosensation (Fig. 2B). Detecting the direction of airflow is critical for long-range odor tracking as it provides important directional cues. Neurons in the antennal lobe can themselves be responsive to airflow through projections of mechanosensory hairs or the responses of ORNs to mechanosensory stimuli (Anton and Hansson, 1994; Galizia et al., 2000; Han et al., 2005). However, the specialized mechanoreceptors for detecting airflow are found in the Johnston's organ in insect antennae (Ai et al., 2007; Kamikouchi et al., 2009, 2006; Schneider, 1964; Yorozu et al., 2009). These receptors are highly sensitive to airflow; *Drosophila* can behaviorally respond to air speeds as low as 0.5 cm s^−1^, a flow rate that is well within speeds described as ‘calm’ by humans (Yorozu et al., 2009). The information from the two antennae are combined to decode the direction of wind (Suver et al., 2019). Flies pick a heading with respect to the direction of airflow and can respond to changes in direction with changes in heading (Currier et al., 2020; Okubo et al., 2020). Nevertheless, work is needed to assess how well insects can disambiguate exogenous airflow from motion-generated airflow. It is also unknown how well insects can assess the mean wind direction in a natural environment with variable wind speed and direction.

Next, we will discuss vision. Two kinds of visual information are important in odor-guided locomotion (Fig. 2C). The first kind is wide-field motion created by self-motion; as the animal moves, the world moves past it. This pattern of movement is critical for controlling speed and assessing whether one is going straight or turning and for stabilizing flight paths (Borst, 2014; Egelhaaf et al., 2012; Srinivasan, 2011, 2014; Taylor and Krapp, 2007). Wide-field information is carried by lobula plate tangential cells (LPTCs) (Fig. 2C). LPTCs project to multiple regions in the brain, including the superior slope, where visual and olfactory information is integrated to generate motor commands. The activity of the LPTCs themselves is modulated by odors (Wasserman et al., 2015); LPTC responses are amplified in the presence of odors, which is likely important for a correct orientation into the wind during the surge. A second type of visual information critical to behavior is the detection of visual features in the environment, such as the long vertical shapes resembling a tree, or detecting a small object as a conspecific. Information about visual features is carried by another set of neurons called the lobula columnar neurons (LCs) (Fig. 2C). A comprehensive analysis in *Drosophila* has revealed that there are 22 LCs that encode different visual features and likely play an important role in olfactory behavior (Wu et al., 2016) that is directed at an object. LCs directly interact with motor pathways and mediate visuo-motor behaviors (Bidaye et al., 2020; Cheong et al., 2020; Namiki et al., 2018a); LC inputs are also integrated with other inputs in the posterior part of the brain. Through mechanisms that are not well understood, neurons downstream of the LCs likely play an important role in integrating visual information about objects with their smell to drive behavior.

Other regions important for odor-guided behavior such as the mushroom body and lateral horn also receive visual inputs (see below). Many different streams of visual information into the central complex, a region of the brain important for computing an insect's spatial orientation, are likely to exist because neurons in the central complex are responsive to different kinds of visual information including self-motion (Hulse et al., 2021; Lyu et al., 2022; Stone et al., 2017). The neural pathways that carry visual information related to self-motion – wide-field visual information such as optic flow – into the central complex are currently unclear, but are under investigation.

### Higher-order olfactory processing and multi-modal integration

PNs from the antennal lobe project to two higher-order processing centers, the mushroom body and the lateral horn (Galizia and Rössler, 2010; Kirschner et al., 2006; Martin et al., 2011; Masse et al., 2009), although minor connections to other protocerebral regions (see Glossary) also exist (Aso et al., 2014b; Tanaka et al., 2012). Both the mushroom body and lateral horn are centers for multi-modal integration and participate in an array of computations through their multimodal input and through connections to other higher brain centers (Fig. 1B).

#### Integration at the mushroom body

The major sensory input into the mushroom body in many insects is from PNs; in flies, only excitatory PNs provide input into the mushroom body, whereas the situation for other insects has not been investigated (Bates et al., 2020). The mushroom body also receives inputs from other sensory modalities, encoding information about temperature (Frank et al., 2015; Liu et al., 2015), humidity (Marin et al., 2020), taste (Kirkhart and Scott, 2015; Masek et al., 2015), visual stimuli (Ehmer and Gronenberg, 2002; Li and Strausfeld, 1999) and mechanical stimuli (Li and Strausfeld, 1999). The relative importance of these inputs depends on the taxa: cockroaches receive more mechanosensory input, whereas bees receive more visual input (Menzel, 2012). These sensory inputs interact with the main local neurons of the mushroom body called the Kenyon cells in a region of the mushroom body called the calyx; the axons of the Kenyon cells project to the lobes, which are segmented into processing units. Each segment receives input from a subset of dopaminergic neurons and outputs to a subset of mushroom body output neurons (Aso et al., 2014a; Strausfeld et al., 2009). The input–output relationship between Kenyon cells that carry input sensory information and mushroom body output neurons that carry output behavioral messages is modified by signals from dopaminergic neurons to affect learning (Martin et al., 2011; Menzel and Giurfa, 2001; Modi et al., 2020). This neural architecture is perfect for associating odors with other events in the world.

However, associating odors with events is not the only role of the mushroom body in odor-guided behavior. Both the dopaminergic neurons and the output neurons interact with premotor circuits and with output neurons from the lateral horn (Aso et al., 2014a; Dolan et al., 2019; Schlegel et al., 2021), and are in the correct place in the circuit to perform sensorimotor transformations including those involved in odor-guided locomotion. When processing in mushroom body is blocked, either by chemical ablation or through genetic methods, it leads to elevated locomotor activity in flies (Martin et al., 1998), crickets and grasshoppers (Huber, 1974). Activating individual mushroom body output neurons can produce attraction or repulsion to odors (Aso et al., 2014b) and also promote upwind movement when activated (Matheson et al., 2022). Similarly, manipulating dopaminergic signaling in the mushroom body of flies can affect movement on a trial-by-trial basis (Handler et al., 2019; Zolin et al., 2021). It has been hypothesized that in a complex environment with multiple odor sources, the mushroom body can tie together inputs from PNs that are activated at the same time, allowing disambiguation of different olfactory stimuli (Baker and Hansson, 2016).

#### Sensory integration at the lateral horn

The circuit architecture of the lateral horn is strikingly different from that of mushroom body. The lateral horns in all insects studied thus far receive inputs from all PNs (Galizia and Rössler, 2010); in flies, this includes the excitatory PNs that also project to the mushroom body and the inhibitory PNs (Bates et al., 2020; Schlegel et al., 2021). The lateral horn also receives input from other sensory modalities, including gustation, mechanosensation, thermosensation and vision (Chakraborty and Sachse, 2021), as well as from the mushroom body (Dolan et al., 2019; Schlegel et al., 2021). Unlike the mushroom body, which is segmented into clear and well-defined processing units, the lateral horn is a diffuse neuropil (Sun et al., 1997; Yasuyama et al., 2003), and the underlying computational logic is not obvious. The connectivity pattern between projection neurons, the intrinsic and output neurons of the lateral horn, is stereotyped enough that the same neurons (similar anatomy, connections and responses) can be identified across animals (Bates et al., 2020; Caron et al., 2013; Jeanne et al., 2018; Jefferis et al., 2007; Schlegel et al., 2021). Based on this connectivity pattern, the lateral horn consists of ∼500 cell types in *Drosophila* compared with only 15 types of Kenyon cells (Schlegel et al., 2021). There are also more than 37 types of output neurons. Although there is some disagreement among different studies, neurons in the same morphological class have similar odor–response profiles (Frechter et al., 2019; Jeanne et al., 2018), once again highlighting the stereotyped nature of the circuit.

There is some evidence that the lateral horn can function as a site for computing odor valence, i.e. whether an odor is attractive or repulsive (Strutz et al., 2014), or as a site for encoding odors based on chemical structure (Frechter et al., 2019). However, there is hardly any consensus regarding the fundamental computations performed in the lateral horn. The lateral horn output neurons project to different regions of the protocerebrum, where they interact with outputs from the mushroom body and with premotor circuits (Schlegel et al., 2021). Given that the lateral horn receives multisensory input from the mushroom body and downstream motor areas, it is unlikely that the lateral horn functions purely as a center for integration of olfactory input (Chakraborty and Sachse, 2021; Martin et al., 2011; Schlegel et al., 2021). This conclusion is supported by a recent comprehensive analysis of the anatomy of the lateral horn in *Drosophila*, which found that many lateral horn neurons receive more feedback input from motor areas than feedforward sensory inputs (Schlegel et al., 2021).

The lateral horn appears to play an important role in many innate behaviors driven by ecologically important stimuli. For example, the behavioral responses of *Drosophila* to CO_2_, which is sensed by a single ORN class, appear to be completely mediated by the lateral horn (Varela et al., 2019); the behavioral response to geosmin, an odor that signals harmful microbes, is another example (Huoviala et al., 2020 preprint). In the context of a moth's behavioral response to pheromones, a region adjacent to lateral horn, often referred to as inferior lateral protocerebrum, is a site where inputs from monoglomerular PNs, multiglomerular PNs and inhibitory PNs are integrated (Anton et al., 1997; Kanzaki et al., 2003; Kárpáti et al., 2008, 2010; Lee et al., 2019). One hypothesis is that this integration is important to differentiate between individual pheromone components versus a blend. Alternatively, different kinetics of the neural response and different axonal lengths of these PNs might provide important information about the stimulus (Lee et al., 2019). In most insects, the lateral horn is also a site for integration of information from the two antennae (Hansson and Stensmyr, 2011). Finally, some of the integration of odor inputs with wind and visual input also occurs in the lateral horn (Baker and Hansson, 2016; Schlegel et al., 2021).

In total, the mushroom body and the lateral horn are not just centers for olfactory integration; rather, they are highly recurrent circuits for sensorimotor transformation. How these two regions of the brain interact with downstream motor circuits to control behavior is an important avenue for future research.

### Circuits integrating spatial information with sensory input to produce motor commands

The spatial context for orientation and navigation is computed in the central complex, which is a collection of central brain neuropils. Many recent reviews describe the computation performed in the central complex (Heinze et al., 2018; Hulse et al., 2021; Pfeiffer and Homberg, 2014; Turner-Evans and Jayaraman, 2016; Webb and Wystrach, 2016). In brief, two of the central complex neuropils, the ellipsoid body and the protocerebral bridge, record the current heading. The central complex also receives direct information related to wind direction (Currier et al., 2020; Homberg, 1994; Matheson et al., 2022; Okubo et al., 2020; Ritzmann et al., 2008), which allows it to reference internal representations to external directional stimuli such as wind direction; insects use the central complex to orient to airflow (Fig. 1B). Silencing fan-shaped body neurons – neurons within a sub-region of central complex – affects the ability of flies to make corrective turns with respect to the wind (Currier et al., 2020).

The lateral accessory lobe receives information regarding both orientation and pheromones (Seki et al., 2005) through medial protocerebral neurons that, in turn, receive input from the lateral horn (Namiki et al., 2014). Many descending neurons (DNs) receive input from the lateral accessory lobe (Fig. 2D). These DNs, therefore, have much of the information needed to send navigation-related motor commands, and many are responsive to pheromones (Kanzaki et al., 1994). An interesting property of these neurons in the moth is that they are bistable; thus, they are referred to as flip-flop neurons (Kanzaki et al., 1994). Each state lasts up to 30 s, with state transitions being mediated by a new stimulus. Thus, these flip-flop neurons have the correct properties necessary to mediate an insect's behavior, including the internally generated counter-turns that are non-reflexive. Pheromone-sensitive DNs also originate from a region of the brain called the posterior slope. These DNs receive pheromone-related information directly from the medial protocerebrum. At least in the case of moth pheromones, these DNs have a phasic response to pheromones (Namiki et al., 2018b) and are likely responsible for mediating stimulus-triggered responses such as the phasic surge response or the turn response to odor.

Much remains to be discovered in terms of which DNs respond to odor stimuli and the relationship between DNs and behavior. Nevertheless, studies seeking to model plume tracking show that turns driven by the flip-flopping neurons can serve as a mechanism for odor tracking (Adden et al., 2022; Ando et al., 2013). In these two studies, outputs of flip-flopping neurons were used to guide turns; two mutually inhibiting flip-flop neurons drive turns on each side of the body. Such a simple system appears to replicate the moth's odor-tracking behavior.

## Identification of odor and identity-dependent behavior

Thus far, our Review has focused on the neural mechanisms involved in locating the odor source. Another important problem is identifying the odor, a task for which the olfactory system is optimized (Box 3). Odor discrimination is essential for associative learning and has been covered in detail elsewhere (Laurent, 2002; Masse et al., 2009; Su et al., 2009; Wilson, 2013).

Odor discrimination is also important for instantaneous behavioral decisions. One theme that has emerged in this regard is that many ORNs are specialists and respond specifically to a single ecologically relevant odor. These odors are important for a range of odor-gated behaviors, such as courtship (Dickson, 2008), aggregation, food avoidance and approach, aggression and choice of substrate for egg laying (Anderson, 2016; Aranha and Vasconcelos, 2018). An important idea is that these specialist ORN classes function as a ‘labeled line’, where they signal to a few dedicated neurons at each processing stage to connect odors to specific behaviors. Recent electron microscopic reconstruction of the *Drosophila* olfactory circuit shows that, particularly at the level of the lateral horn and beyond, the signals from the specialist ORN classes diverge to many downstream neurons (Huoviala et al., 2020 preprint). This divergence makes sense because most ecologically important behaviors are both multimodal and plastic – properties that require extensive integration.

Moth sex pheromones are also specialist odors. A major component of most moth pheromones, bombykal (Baker and Hansson, 2016), activates a single ORN type with high specificity. In many moth species, odor-tracking behavior is elicited by a specific blend of odors in the correct ratio rather than by a single compound (Baker, 2008; Berg et al., 2014; Mustaparta, 1997; Vickers et al., 1991; Vickers, 2002), a characteristic that is important in ensuring that a male is tracking only its conspecific. One question is whether a moth waits for the exact blend or whether aspects of the behavior can be triggered by a non-optimal blend. Existing data suggest that even in moth species in which the full tracking program relies on the exact blend, this requirement is less stringent for certain aspects of the behavior, such as initiation of upwind flight (Vickers, 2002). Moreover, addition of pheromone components from a closely related species affects some aspects of the tracking motor program (Mustaparta, 1997; Vickers, 2002; Wu et al., 2015) while leaving others intact. These data suggest that odor tracking is not organized as a unitary behavior; rather, it is a result of parallel sensorimotor loops that connect activity in known ORNs to aspects of the overall behavior.

The question of whether odor modulation of locomotion is composed of independent sensorimotor loops was addressed in targeted experiments designed to ask how different combinations of active ORNs affect a fly's locomotion (Jung et al., 2015). The authors created an arena in which a known combination of ORNs could be activated, and found that each ORN class only affects a subset of locomotor behaviors. These results are best interpreted as a sensory-motor transformation between active ORN classes and the eventual behavior. As an example, they found that activating just one ORN class – one containing the *Or42b* receptor – affects the run duration. However, a combination of multiple active ORNs is essential to change the propensity to turn sharply. Thus, each combination of active ORN classes can be thought of as a sensory-motor feature that affects a particular aspect of locomotion, a conclusion supported by another recent study (Matheson et al., 2022). The olfactory circuits – particularly those in the lateral horn – are tailor-made to make these sensory motor transformations.

## Conclusions and future work

Over the last few decades, much progress has been made in discovering the behavioral algorithms that underlie insects' behavioral response and their neural implementation. This progress provides a strong framework with which gaps in our knowledge can be approached.

One deficit is the absence of the complete dataset required to understand olfactory behavior in nature: simultaneous tracking of the position of the animal along with the odor stimulus, wind direction and other sensory signals. With modern techniques to locate an insect's position (Knight et al., 2019) and wireless electronics to sense the environment and measure electrical signals in real-time (Harrison et al., 2011; Pawson et al., 2020; Thomas et al., 2012), it seems possible to study odor-guided locomotion in a natural environment, particularly in the context of large insects. These datasets, when combined with modern statistical methods (Datta et al., 2019) for analyzing behavior and the relationship between neural responses and behavior, have the potential to not only illuminate odor-guided locomotion in detail, but also to contribute immensely to our understanding of the inner workings of the brain.

Another rich area for future work is understanding the neural implementation of odor-guided behaviors in the brain. Here, recent progress in *Drosophila* in generating genetic tools to probe specific neurons (Luan et al., 2020), to activate and inactivate neurons (Simpson and Looger, 2018), as well as large-scale datasets (Dorkenwald et al., 2021) that describe connectivity between neurons in the brain, enable progress in understanding the sensorimotor transformation at the level of single neurons. Finally, great strides have been made in introducing genetic tools in other insects (Mansourian et al., 2019).

In summary, we predict a productive future for a comparative approach to understanding odor-guided locomotion using large insects in field studies, through leveraging the power of genetic tools and neuroanatomy in *Drosophila* and, finally, through the introduction of powerful genetic tools across other insect species.
